# Smartphone as a Disease Screening Tool: A Systematic Review

**DOI:** 10.3390/s22103787

**Published:** 2022-05-16

**Authors:** Jeban Chandir Moses, Sasan Adibi, Nilmini Wickramasinghe, Lemai Nguyen, Maia Angelova, Sheikh Mohammed Shariful Islam

**Affiliations:** 1 School of Information Technology, Deakin University, Burwood, VIC 3125, Australia; jcmoses@deakin.edu.au (J.C.M.); sasan.adibi@deakin.edu.au (S.A.); maia.a@deakin.edu.au (M.A.); 2 Iverson Health Innovation Research Institute, Swinburne University of Technology, Hawthorn, VIC 3122, Australia; nwickramasinghe@swin.edu.au; 3 Department of Information Systems and Business Analytics, Deakin Business School, Deakin University, 221 Burwood Highway, Burwood, VIC 3125, Australia; lemai.nguyen@deakin.edu.au; 4 Institute for Physical Activity and Nutrition (IPAN), Deakin University, Burwood, VIC 3125, Australia

**Keywords:** technology, smartphone applications, disease screening, mobile solutions, technology acceptance, chronic disease

## Abstract

Disease screening identifies a disease in an individual/community early to effectively prevent or treat the condition. COVID-19 has restricted hospital visits for screening and other healthcare services resulting in the disruption of screening for cancer, diabetes, and cardiovascular diseases. Smartphone technologies, coupled with built-in sensors and wireless technologies, enable the smartphone to function as a disease-screening and monitoring device with negligible additional costs and potentially higher quality results. Thus, we sought to evaluate the use of smartphone applications for disease screening and the acceptability of this technology in the medical and healthcare sectors. We followed a systematic review process using four databases, including Medline Complete, Web of Science, Embase, and Proquest. We included articles published in English examining smartphone application utilisation in disease screening. Further, we presented and discussed the primary outcomes of the research articles and their statistically significant value. The initial search yielded 1046 studies for the initial title and abstract screening. Of the 105 articles eligible for full-text screening, we selected nine studies and discussed them in detail under four main categories: an overview of the literature reviewed, participant characteristics, disease screening, and technology acceptance. According to our objective, we further evaluated the disease-screening approaches and classified them as clinically administered screening (33%, n = 3), health-worker-administered screening (33%, n = 3), and home-based screening (33%, n = 3). Finally, we analysed the technology acceptance among the users and healthcare practitioners. We observed a significant statistical relationship between smartphone applications and standard clinical screening. We also reviewed user acceptance of these smartphone applications. Hence, we set out critical considerations to provide equitable healthcare solutions without barriers when designing, developing, and deploying smartphone solutions. The findings may increase research opportunities for the evaluation of smartphone solutions as valid and reliable screening solutions.

## 1. Introduction

Chronic diseases, such as diabetes, cardiovascular disease (CVD), cancers, and chronic lung diseases are growing exponentially [1]. Disease screening identifies diseases at an early and pre-symptomatic stage by applying tests, examinations, or other procedures that can be applied rapidly [2]. Consequently, it is possible to effectively prevent or treat the identified disease with appropriate medical intervention for those at risk [2,3], thereby ensuring minimal disease and treatment burden to the patient and minimising costs to the healthcare system. The prescribed screening test needs to be reliable and accurate since false negatives or false positives could worsen the health outcomes and incur unnecessary diagnostic costs. In addition, the screening tool needs to be accessible by all income groups [3]. Thus, a screening tool should be demonstrably simple, valid, reliable, quick to administer, cost-effective, and easy to use [2].

The healthcare system plays a pivotal role in screening and subsequent medical procedures to improve the individual’s quality of life, i.e., assist in living healthy with minimal health complications [4]. However, due to the subtle nature of COVID-19 and the stringent guidelines that must be adhered to while visiting hospitals globally, there has been reluctance among people to visit hospitals for screening, diagnosis, routine check-ups, and other healthcare services, which consequently could result in long-lasting health implications [5]. Furthermore, COVID-19 halted cancer screening programmes in the United Kingdom, prejudicing the chances of early diagnosis [6], which might have better health outcomes when compared with detecting cancer at an advanced stage. Moreover, adults postponed or cancelled their hospital appointments to screen for diabetes and dyslipidemia during the COVID-19 pandemic [7]. Furthermore, there was a decline in the preventive screening and monitoring of chronic diseases, such as lipid disorders and diabetes [7]. The lack of screening and monitoring of diabetes could result in dangerously out-of-control levels, resulting in other health impairments [7]. The reduction in physical activities due to strict social distancing policies further strengthens the importance of early disease screening for people with high risk, e.g., those over 65 years of age.

The innovation in smartphone technologies, coupled with built-in sensors and wireless technologies, enables the smartphone to function as a device for disease screening and monitoring with negligible additional costs [8]. Recent reviews have evaluated the role of apps in supporting self-management and transition amongst chronically ill young people, improving chronic patients’ lifestyles, and lifestyle improvement in non-communicable diseases [9]. One review exclusively explored app features with outcomes in studies involving chronic respiratory diseases, diabetes, and hypertension [10]. To our knowledge, there is no recent literature review available that evaluates the use of apps to screen for chronic diseases in an adult population. Thus, this paper aims to address this gap to evaluate the use of apps in disease screening. Here, we evaluated the acceptability of apps as a screening tool by various stakeholders in medical and healthcare systems to assess the criteria to be considered when designing, developing, and deploying apps.

## 2. Materials and Methods

Our systematic review followed the Preferred Reporting Items for Systematic Reviews and Meta-Analyses (PRISMA) guidelines [11], and, since the primary investigators obtained informed consent, ethics approval was unnecessary [8].

### 2.1. Data Sources and Search Strategy

We performed the literature search in online database publishing articles on health and medical research published in English, including Medline Complete, Web of Science, Embase, and Proquest. The search terms were ‘mhealth OR mobile health OR m-health OR mobile app OR mobile application OR smartphone application OR app OR apps’, AND ‘disease OR illness OR sickness OR condition OR disorder OR health’, AND ‘screening OR assessment OR test OR diagnosis’. With several apps available for chronic disease management, but lacking effectiveness evidence [9,12], the World Health Organisation recommended rigorous evaluation in research settings to analyse the benefits, harms, acceptability, feasibility, resource use and equity considerations of digital health interventions before deployment [12], leading us to consider articles evaluated and published in research journals. We represent our final search in Table S1 following Medline search, and we adapted this to the specifications of the other databases accordingly [8].

### 2.2. Study Selection Criteria

We limited the search to between January 2010 and September 2020, applying the selection criteria described in Table 1, matching the period of disruptive innovations in wireless technologies, such as Bluetooth, ZigBee, and 4G, and app development for health applications (mHealth). We specifically aimed to evaluate the role of apps in disease screening among adults and hence considered participants aged 18 and above, as adulthood is considered to be reached by the age of 18 [13]. Most studies are at a prototype stage; hence, to obtain credible results, we considered studies with at least 15 participants [14].

### 2.3. Study Selection Process

**Figure 1:**
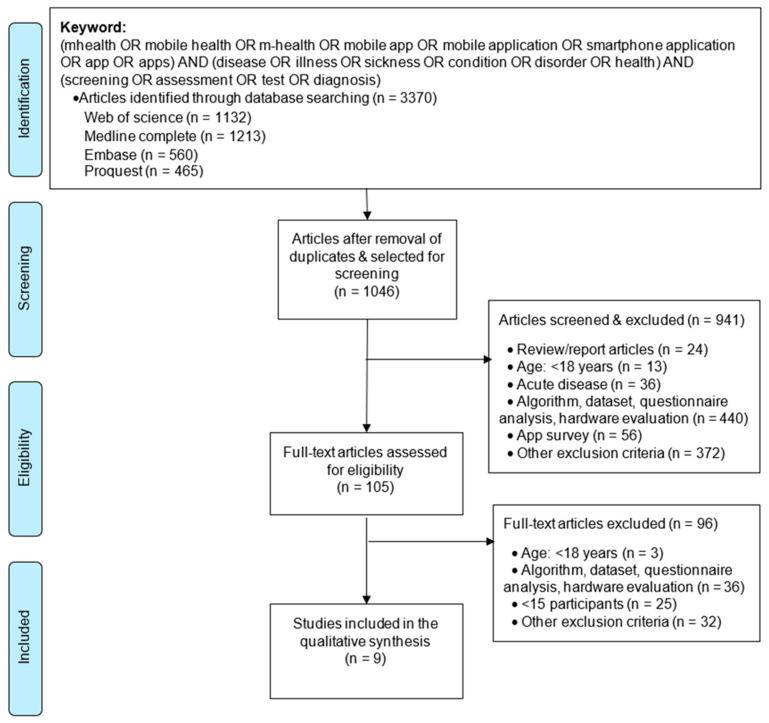
Flow diagram for selection of articles (adapted from [11]).

We illustrate the article selection process in Figure 1. We imported the citations (n = 3370) into the reference manager software EndNote and eliminated duplicates, which resulted in 1046 articles. We then retrieved the title and abstract of all papers (n = 1046) electronically, and we applied selection criteria to eliminate publications inconsistent (n = 941) with the objective of this review. Matching the study’s objectives, we examined the full texts of the remaining papers (n = 105) and selected nine papers (n = 9) for our study that reported an app-based disease-screening system for the adult population.

### 2.4. Data Extraction and Analysis of Selected Studies

A reviewer evaluated the titles and abstracts of all records identified in the initial database search and then assessed the full text for eligibility according to the inclusion criteria. In addition, a second experienced reviewer oversaw the process, and after the article evaluation and extraction, the reviewers discussed and reviewed the selection. Then, we extracted data regarding the study settings and participant details. We further gathered details regarding the mHealth technology used and the methodology adopted to perform the screening. We considered the outcomes of each study, presenting the primary outcomes and their statistically significant value with the cut-off point of the results as designated by *p*-values at *p* < 0.05 [8]. Further, we examined the user acceptance of the deployed technology and have presented the findings.

## 3. Results

### 3.1. An Overview of the Literature Reviewed

Table 2 summarises the study details and participant characteristics. 

**Figure 2:**
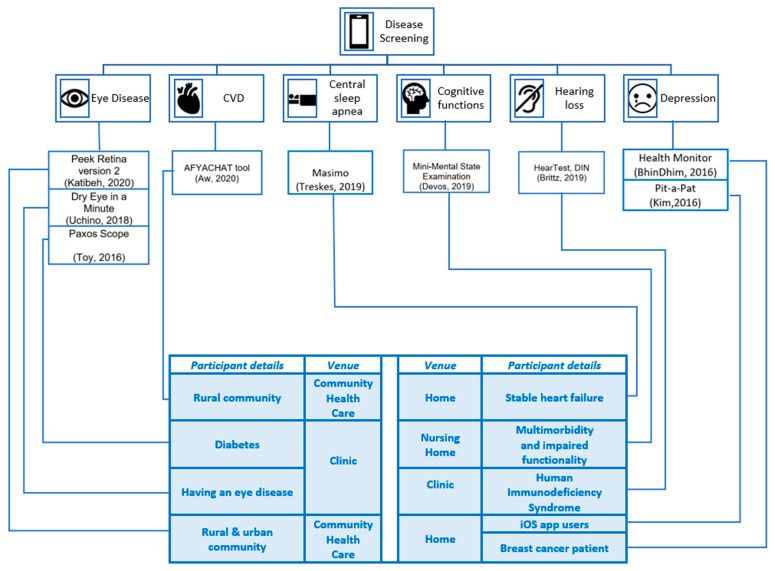
Disease-screening apps screen eye disease, cardiovascular disease, central sleep apnoea, cognitive functions, hearing loss, and depression.

Several apps were used in the studies to screen for disease by recording user perceptions [18,22,23], capturing physiological recordings [16,17], and obtaining clinical readings/recordings [15,19,20,21]. The number of study participants varied between fifteen [18] and over two thousand [15,22]. The studies were experimental [15,19] and observational [16,17,18,20,21,22,23]. Table 3 represents the countries in which the studies took place and the economic status and mobile cellular subscriptions per 100 people; the latter show an upward trend in cellular subscriptions over the years [24,25]. Figure 2 illustrates the app functionalities to screen chronic illness, such as CVD [16], central sleep apnoea [17], cognitive impairment [18], depression [22,23], eye disease [15,20,21], and hearing loss [19]. In a single session, screening for CVD, eye disease and hearing loss were performed [15,16,19,20,21]. To screen for central sleep apnoea, cognitive impairment, and depression, multiple recordings/data entries were obtained [17,18,22,23].

### 3.2. Participant Characteristics

The medical conditions of the study participants varied across the studies. For example, the studies screening for eye disease, CVD, and depression recruited undiagnosed people from rural and urban communities [15,16,22]. In contrast, other studies enrolled participants living with chronic diseases, such as stable heart failure (HF) [17], multimorbidity and impaired functionalities [18], human immunodeficiency virus (HIV) [19], eye disease [20], diabetes [21], and breast cancer [23]. However, the magnitude of disease among the participants differed. A study amongst diabetic patients living with diabetes for 11.9 ± 8.4 years screened for diabetic eye disease [21]. Another study screening patients for dry eye disease (DED), a multifactorial disease characterised by unstable tear film, considered participants having an eye disease [20]. Other studies considered patients visiting clinics for their health and wellbeing, including HIV patients [19], breast cancer patients [23], and HF [17]. In another study, the app screened older adults residing in nursing homes for cognitive impairment [18].

#### Age

The age of the participants varied between 18 and 84 [15,16,17,18,19,20,21,22,23]. A single study’s most extensive age range was between 24 and 84 [20]. The highest age group in a study comprised elderly participants aged over 80 [18]. Studies reported associations between the magnitude of disease and age. For example, clinical evaluation of hearing loss using automated audiometry hearing health technology for HIV patients between 18 and 55 years (mean age: 44.4 years) did not detect any significant variation in high-frequency hearing loss among the study participants [19]. However, the prevalence of DED was significantly higher (*p* = 0.04) among the older population (age: 55.2 ± 3.4 years) compared to the younger population (age: 48.1 ± 2.7 years) [20]. A statistically significant trend toward decreasing visual acuity with increasingly severe photograph grades of retinopathy (*p* = 0.046) was observed across older age group participants [21]. Similarly, ageing was associated with increased eye care utilisation due to diabetes, vision problems, hereditary visual impairments, and other medical conditions [15]. Moreover, participants aged over 65 years had a higher prevalence of systolic blood pressure (SBP > 140 mmHg, 41% vs. 25%, OR 2.1, 95% CI: 1.6–2.9, *p* < 0.0001) and self-reported diabetes (4.3% vs. 1.7%, OR 2.5, 95% CI: 1.2–5.4, *p* = 0.01) when compared with younger participants [16]. In addition, age was positively correlated with SBP (ρ = 0.16; *p* < 0.0001) and was observed to be a major CVD risk factor [16].

### 3.3. Disease Screening

**Figure 3:**
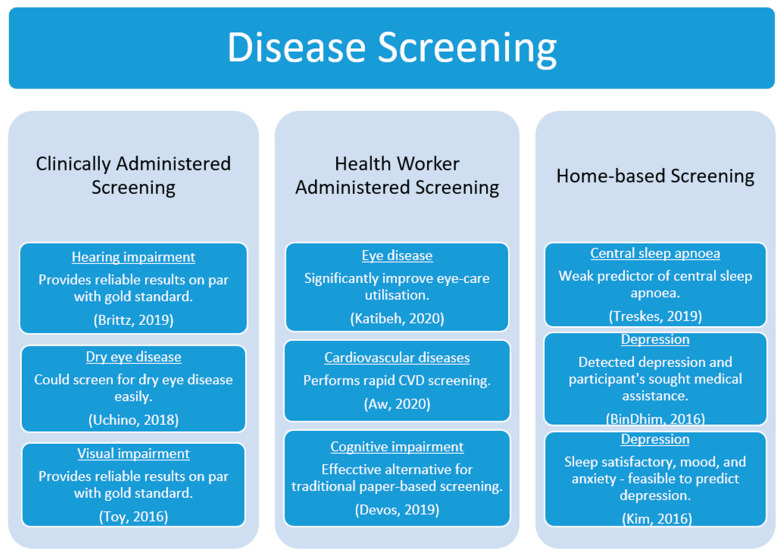
Disease screening and significant outcomes.

Screening aims to achieve early detection of the disease as part of primary prevention or effective treatment in people before the occurrence of the disease or any symptoms of the disease. The screening methodology varied between the studies. For example, screening for comorbidities, such as hearing and visual impairments, amongst HIV and diabetes patients visiting clinics, were performed [19,21]. People with eye disease visited the clinic to screen for DED [20]. Trained health workers visited homes and/or invited residents to community centres to screen for eye diseases and CVD [15,16]. Similarly, health workers caring for the elderly in a care home screened their residents for cognitive impairments [18]. Moreover, central sleep apnoea and depression screening was self-administered at the participant’s residence [17,22,23]. Accordingly, we have categorised the studies based on the screening methodology administered as clinically administered screening [19,20,21], health-worker-administered screening [15,16,18], and home-based screening [17,22,23]. Figure 3 represents the screening apps, the administered methodology, and significant outcomes.

#### 3.3.1. Clinically Administered Screening

Hearing impairment is closely associated with many infectious diseases (IDs), including HIV, due to intrinsic causes related to the infection and extrinsic causes related to the medications. Hearing assessment apps were used in clinical settings to record audiometry data as well as speech recognition threshold (SRT) amongst the HIV-infected participants and were validated against standard clinical testing devices (otoscopy: Welch Allyn 719 Series lithium-ion power handle otoscope, and tympanometry: Grason–Stadler tympanometer). The hearing assessment apps detected hearing loss amongst 53% (n = 106) of the participants, whereas standard procedures could detect it amongst 48% (n = 96) of the participants. A strong positive correlation between hearing assessment apps and standard procedures was found, with correlation coefficients ranging between 0.76 and 0.79. A moderate to strong positive correlation (0.44 to 0.88) across all test-retest threshold readings was observed using the apps. The validated results obtained in clinic settings were used as a baseline reading and monitoring tool at the ID clinics [19].

Prolonged video display terminal (VDT) usage is a leading cause of DEDs amongst the other causative factors for the disease. The clinical evaluation of DED comprised initial questionnaire-based screening for DED symptoms followed by objective verification of the condition through a tear function test and ocular evaluation process, such as standard tear-film breakup time (TBUT) and Meibomian gland dysfunction (MGD) grading. The app verified the subjective questionnaire-based responses recorded for functional visual activity (FVA) to measure the participant’s ability to identify the Landolt ring’s appearance on the screen by pressing the arrow on the smartphone. It measured the proportion of instances of correct identification of the ring, with gradual decrease in the ring’s size, whereas failure to identify the ring resulted in the ring increasing in size. On evaluation, the FVA maintenance ratio measured by the app showed a significant difference between subjects with and without DED (0.76 ± 0.04 vs. 0.91 ± 0.55, *p* < 0.0047). Significant associations were also found between the apps DED evaluation and the questionnaire score (r = 0.39, *p* = 0.002), as well as between the apps DED evaluation and the TBUT result (r = −0.30, *p* = 0.018) [20].

Diabetic retinopathy and diabetic macular edema are common ophthalmic sequelae of diabetes. All patients underwent comprehensive ophthalmic examination, including spectacle-corrected distance visual acuity on conventional Snellen charts, slit lamp examination, and dilated ophthalmoscopy, and concurrently underwent smartphone-assisted acquisition of spectacle-corrected near visual acuity and anterior/posterior segment photography. A good correlation between clinical Snellen and smartphone visual acuity measurements (ρ = 0.91) was observed. Similarly, app-acquired fundus photographs demonstrated 91% sensitivity and 99% specificity to detect moderate non-proliferative and worse diabetic retinopathy, with good agreement between clinic and photograph grades (kappa = 0.91 ± 0.1, *p* < 0.001; AUROC = 0.97, 95% CI: 0.93–1) [21].

#### 3.3.2. Health-Worker-Administered Screening

Participants were randomly classified into mobile screening group (vision test performed using a smartphone) and conventional screening group (vision test performed using fundus photograph equipment (Visucam Pro NM; Carl Zeiss Meditec AG, Jena, Germany) and Snellen charts). On following up after the initial screening, a significant improvement was observed in the eye care utilisation (OR = 1.5, 95% CI: 1.2–1.9) among the referred participants, and this improvement was more significant in the mobile screening group (OR:1.7, 95% CI: 1.2–2.4) compared to the conventional group (OR: 1.2, 95% CI: 0.8–1.8) [15].

To assess the CVD risk of participants, age, sex, smoking status and diabetes history, with device-recorded SBP and blood sugar levels were entered on the health workers cellular device and sent as a single short message service (SMS) to the centralised tool. The 10-year CVD risk was computed using the WHO and the International Society of Hypertension (WHO/ISH) risk prediction chart, and patients with CVD risk greater than 10% were followed-up. Among patients with self-reported diabetes, SBP was higher (52.9% vs. 26.4%, OR: 3.1, 95% CI: 1.6–6.1, *p* = 0.0005) compared to non-diabetes patients. Furthermore, 98% (n = 1611) had a 10-year CVD risk less than 10%, whereas 2% (n = 39) had a risk greater than 10% [16].

Care staff in a nursing home administered a paper-based Mini Mental State Examination (MMSE) questionnaire and an app-version of a slightly modified MMSE questionnaire to elderly residents for five months to assess their cognitive function. A 74% similarity in paper-based and app-based MMSE responses was found. Furthermore, on computing the final test score (maximum 30), a positive correlation (r = 0.90, *p* < 0.01) was observed between paper-based and app-based MMSE responses [18].

#### 3.3.3. Home-Based Screening

Depression screening in home settings was undertaken [22,23]. A study amongst breast cancer patients obtained daily mental-health ratings (sleep satisfaction, mood, and anxiety) and compared them against bi-weekly PHQ-9 ratings over 48 weeks to evaluate the potential of mental-health ratings to be used as an indicator for mental health. On evaluation, it was found that the ROC curve (AUC) was 0.8012, indicating that mental-health ratings were comparable to PHQ-9 results and could be used as a tool for screening for depression in practice [23].

Another study examined the role of depression-screening apps in motivating users with high depressive symptoms to seek healthcare among Apple app users (n = 2538) in five countries (Australia, Canada, New Zealand, the United Kingdom, and the United States). The participants completed PHQ-9 screening, and those obtaining a score of 11 or above were diagnosed as depressed. The study observed high depressive symptoms among 29% (n = 741) of whom 419 participants were previously diagnosed with depression. On following up after a month with the participants diagnosed with depression (n = 322) through this screening, it was found that 38% (91/238) of participants consulted their healthcare professionals about the depression score provided via the app, signifying that there was an increased follow-up response rate (OR 3.2, 95% CI: 1.38–8.29) [22].

A study was undertaken for four nights to assess the feasibility of a smartphone-compatible oximeter to detect central sleep apnoea in a stable heart failure participant group. The oximeter recordings were compared against the polygraph recordings obtained on the first night of the study, and a difference of 2.1 for the higher oxygen desaturation index (ODI) was observed in the oximeter compared with the polygraph measure (IQR: −4.3 to 14.3). Similarly, the median saturation yielded by the oximeter was 3.1 (IQR: 2.6–4.1) percentage points higher than that measured by the polygraph. Hence, this implies that ODI measured by oximeter was a weak predictor of central sleep apnoea in patients with stable heart failure [17].

### 3.4. Technology Acceptance

Studies have used various methods to evaluate the acceptance or comfortability of technology used for disease screening. The evaluation process for disease screening technology used to identify DED recorded a satisfactory level among 69% of the participants [20]. The satisfactory level dropped to 47% among the same cohort when offered treatment with an ophthalmologist [20]. Moreover, the participants accepted the CVD screening well, as all the six feedback questions received a favourable response [16]. Furthermore, the significance of the interface was justified in the study undertaken to evaluate the scope of app-based cognitive function screening among the elderly (age: 86.5 ± 5.95 years) [18]. Although the findings suggested that the readings obtained in the digital version were comparable to the standard paper-version readings, the participants preferred devices with a large screen (e.g., tablets) [18]. Furthermore, a study (participants: 26, age: 62–70 years) found that the oxygen desaturation index measured by the oximeter was a weak predictor of central sleep apnoea [17]. Nevertheless, the study was affected by insufficient diagnostic quality, including participants unwilling to undergo a second polygraph examination (n = 2), forgetting to attach the oximeter on their first night (n = 2), failing to email at least a day’s recording (n = 13), and ambiguity in the device attachment methodology [17]. Current research is limited to user acceptance and there is a lack of evidence on how smart applications are integrated and assimilated within clinical care processes, including telehealth. We found no evidence for integrating patient clinical/health records in the reported apps.

## 4. Discussion

Disease screening identifies a disease early in an individual/community to prevent or effectively treat the condition [2,3]. With approximately one in three adults globally suffering from multiple chronic diseases, screening for chronic emerging and remerging diseases is imperative. Furthermore, developing countries face the challenge of chronic diseases, such as CVD, diabetes, chronic respiratory diseases, cancers, mental illnesses and injuries, and a continued burden from communicable diseases [26]. Moreover, infrastructure deficiencies, hospital resources centred in urban areas, and shortage of trained healthcare workers are barriers to equitable healthcare access in developing countries [26]. In addition, the current COVID-19 pandemic has restricted hospital visits for screening, diagnosis, routine check-ups, and other healthcare services, resulting in the disruption of screening for diseases such as cancer, diabetes, and dyslipidemia [5,6,7]. There is widespread use of smartphones globally that can record user values, with built-in sensors able to capture physiological parameters at negligible additional cost [27]. The potential benefits of smartphones could enable them to function as screening tools that could be demonstrably simple, valid, reliable, quick to administer, cost-effective, easy to use, and accessible to all income groups [2,3,27]. Hence, this review aimed to evaluate the use of apps in the disease-screening system and the acceptability of technology in the medical and healthcare sectors.

Our study considered both experimental [15,19], and observational [16,17,18,20,21,22,23], studies that were conducted in economically developed [17,18,20,21,22] and developing countries [15,16,19,23]. Irrespective of the economic levels of the country, there has been an increase in mobile cellular subscriptions per hundred people (Table 3) in developing countries [24]. Furthermore, 5G subscriptions are forecast to reach 2.8 billion globally by the end of 2025, accounting for about 30 per cent of total mobile subscriptions, and the uptake is expected to be significantly higher than it was for 4G [28]. Furthermore, 5G, in conjunction with other emerging technologies, such as the Internet of Things (IoT), artificial intelligence, and big data and data analytics, could offer smart, personalised healthcare solutions to overcome current healthcare barriers [29]. Hence, it is important to design, develop, and deploy mHealth solutions that are secure, reliable, socially acceptable, and can be used by different user groups, including all genders, adults, especially the elderly, and people from all socio-economic backgrounds, to access the system effortlessly.

The age of the study participants varied between 18 and 84 years [15,16,17,18,19,20,21,22,23]. Age was positively correlated with eye disease [15,20] and SBP, a major CVD risk factor [16]. Moreover, the healthcare needs of adults as they age will be demanding, and hence it is essential to design mHealth solutions with appropriate and straightforward interfaces that are easily accessible. Accordingly, usability considerations appropriate to the targeted user’s age group, such as screen size, font size, convenient user interface, and easily attachable hardware devices, should be considered when designing and developing mHealth solutions [17,18]. Furthermore, since older adults are highly prone to chronic diseases, the mHealth solutions should have a barrier-free user interface, so that participants are willing and able to use it routinely, and the system should be tailored to the patient population [17,30]. However, some studies did not evaluate user/technology acceptance [15,17,19,21,22,23], whereas other studies used customised questionnaires to evaluate different user/technology acceptance parameters [16,18,20]. The technology acceptance model (TAM) is widely used to evaluate the user acceptance of an information system [31]. A recent study exploring the acceptance of technology using TAM among older adults with multiple chronic conditions found that older adults used the available technology features in online portals and were motivated to use information systems [32].

For screening administered in-clinic to screen for hearing impairment, visual impairment, and dry eye disease, it was observed that app-based screening provided reliable results on a par with gold standards [19,20,21]. Although the results obtained were clinically accepted, the studies had methodological weaknesses, such as non-randomised subjects, small sample size, and subject selection bias [19,20,21]. Future studies addressing these limitation could enhance the functionality of the developed app. Moreover, studies have found that viruses could be a causative agent for congenital hearing loss (e.g., cytomegalovirus, Rubella, lymphocytic choriomeningitis virus), or may be genetic and acquired (e.g., HIV, herpes simplex virus), or acquired (e.g., measles, varicella-zoster virus, mumps, West Nile virus) [33]. Apps could screen the patients with acceptable accuracy [19]. Although, mHealth benefits healthcare practitioners by providing sophisticated tools, in medical practice only reliable and valid solutions should be considered.

The risk of multimorbidity increases among chronic patients, especially the elderly (≥65 years), increasing healthcare demands [34]. For example, diabetes increases the risk of macrovascular diseases, a disease of any large blood vessels in the body, such as CVD and stroke, by two to four times compared with non-diabetes [35]. In addition, diabetes patients are prone to microvascular diseases, including diabetic retinopathy, diabetic nephropathy, and others [35]. One study undertaken with diabetes participants (n = 50, age: 60.5 ± 10.6 years, diabetes duration: 11.9 ± 18.4 years) visiting an eye clinic for monthly diabetic eye disease screening, observed that there was a decline in visual acuity, signifying an increased severity of diabetic retinopathy [21]. Moreover, diabetes was a significant cause of increased eye care utilisation among participants (61.7 ± 9.5 years) who needed eye care [15]. Furthermore, patients with self-reported diabetes over 65 years had a higher SBP than non-diabetes participants [16]. Although the study objectives, participants’ selection criteria and characteristics, and study settings were heterogeneous, there was an increasingly proportional relationship between diabetes severity and other complications, such as eye disease and SBP, which might, in turn, escalate the risk of CVD [15,16,21]. Furthermore, since there is no cure for diabetes, the management of diabetes involves lifestyle changes and regular monitoring and management by patients and health professionals [36]. Hence, with the advancements in technology, there is an imperative need to develop and integrate non-invasive self-management support tools for screening and monitoring diabetes [36]. Moreover, there is a need to develop solutions that could screen/predict multimorbidity. The COVID-19 global health crisis has induced negative psychological responses, such as anxiety, depression, and increased self-reported stress [37]. The review observed that screening for mental health (n = 2538, five countries) had identified high depressive symptoms among participants who had never been diagnosed with depression (n = 322), and few of them had consulted their healthcare professional [22]. In addition, health parameters from mental-health trackers, such as sleep satisfaction, mood, and anxiety were good predictors of depression [23]. The findings of the study could be applied in these unprecedented times by health practitioners to screen and maintain the mental health of their patients.

### 4.1. Implications for Practice

Design considerations play a vital role in developing mHealth solutions tailored to the needs of the participants, especially for older adults [17,18]. The study suggests that apps could screen diseases accurately according to current gold standards [15,16,18,19,20,21,22,23]. Apart from two apps available in both the app store and Google play store [17,23] and a system functioning on text-based service [16], the other apps used in disease screening were deployed in either the app store [17,21,22] or the Google play store [15,18,19]. Since the global market is poised for rapid growth and could have widespread implications in delivering personalised healthcare [24,28,29], interoperable mHealth solutions should be designed and developed [30].

### 4.2. Implications for Research

The review suggests four implications for research:

1. The effective use of apps in disease screening should be evaluated further by integrating app-based solutions within a clinical screening process/workflow to assess the systems’ clinical accuracy, reliability, and acceptability [19,20,21].
2. User experience should be considered during design. Patient and clinician engagement is needed in the cycles of design and validation to ensure their needs are understood and addressed [17,18].
3. It is premature to precisely predict the sequelae of COVID-19 on physical, psychological and neuropsychological outcomes, and social behaviour [38]. Hence, a literature search to find the potential consequences of the virus and screening technologies currently available for the consequences would be of assistance to the healthcare community and COVID-19 infected/recovered patients to identify symptoms at an initial stage.
4. Our future work is focused on exploring and realising the potential of artificial intelligence (AI), such as predictive analytics/machine learning and image recognition in disease screening and national language processing and conversational AI in disease-screening literacy.

#### Future Research Directions

**Figure 4:**
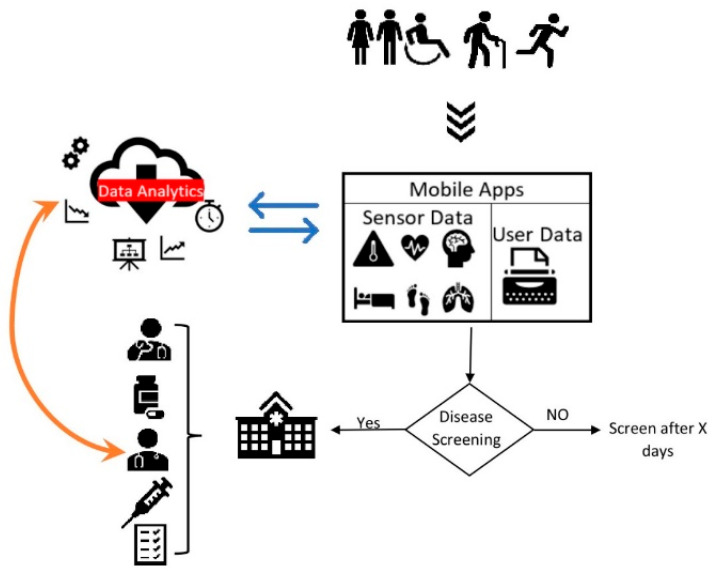
mHealth screening process (illustrated from reference [15,16,22,39,40,52]).

The study has revealed that screening for disease in a community could detect people at risk and motivate at-risk participants to seek further medical attention [15,16,22]. Moreover, we observed an increasingly proportional relationship between chronic diseases, such as diabetes severity and other complications, such as eye disease and SBP, which might, in turn, escalate the risk of CVD [15,16,21]. The multimorbidity complications associated with chronic diseases, such as CVD and diabetes, imply a need to develop mHealth systems that could screen/predict for associated multimorbidity complications. It is anticipated that big data analytics could assist in delivering personalised healthcare [39,40]. Figure 4 illustrates the mHealth screening process and data analytics integration. Research prospects are there for the design, development, and deployment of mHealth systems integrated with AI (such as machine learning/data analytics, conversational agents, machine vision) and IoT. Furthermore, as clinical validity has been established, to examine the uptake, continuance, and significance of the system in a global setting, the apps shall be evaluated using well-tested theoretical models such as TAM, UTAUT [41], the information systems success model [42], and effective use [43]. These theories can guide researchers in evaluating consumer acceptance and satisfaction, and the effective use and perceived impact of technology innovations. We also call for further research into practical use, user experience, and benefits evaluation of smartphone applications for disease screening and monitoring.

Heart rate variability (HRV) is a measurable reflection of the balance between sympathetic and parasympathetic tone [44] and could be used as a marker for predicting diseases. Among CVD patients, lower HRV is associated with a higher risk of cardiovascular events and mortality [45] and could distinguish congestive HF patients from healthy adults [46]. Similarly, HRV parameters may have utility as biomarkers for stroke and post-stroke complications and functionality [47]. In addition, there is an association between HRV and BP [48]. A large prospective international clinical study observed that short-term HRV could be used to assess risk in low- to intermediate-risk individuals without known coronary artery disease [49]. Finally, amongst COVID-19 patients, higher HRV predicts greater chances of survival, and low HRV predicts ICU indication and admission in the first week after hospitalisation [50]. Remote measurement technologies, such as smartphones, wearable sensors, or home-based devices, can capture HRV and could potentially function as an adjunct digital biomarker that could aid in the evolution of current “diagnose and treat” care models [51]. Hence, there are research prospects for the capture of HR in real-time from wearable devices and application of machine learning to screen for chronic disease.

### 4.3. Limitations of This Survey

Our study has several limitations. Although mHealth has found widespread application in disease prevention, our study represents a small proportion of the disease screening process. Secondly, studies had a sample size of less than 100 [17,18,20,21,23] and were undertaken in developed and developing countries. Hence, although the findings could be extended to different populations and larger sample sizes to validate the results’ accuracy, the current findings might not be generalisable. Thirdly, there could be a possibility of bias as one reviewer performed the article screening and data extraction. Fourthly, our timeframe was limited to September 2020. It will be worth performing a similar search from September 2020 to evaluate the role of mHealth systems for disease screening for various chronic diseases since the effect of the pandemic is outside the scope of this study. Finally, data heterogeneity and lack of standard models to evaluate the user perception of the information system have prevented us from conducting a meta-analysis.

## 5. Conclusions

We evaluated the use of apps in disease screening and observed a statistically significant relationship between the app and standard clinical screening methodology recordings. We set out critical considerations when developing mHealth solutions to provide equitable healthcare solutions without barriers. Furthermore, the findings might increase research prospects for the evaluation of mHealth solutions as valid and reliable screening solutions.

## Figures and Tables

**Table 1:** Study selection criteria (adapted from [8]).

Inclusion Criteria:
1. Smartphone applications used and disease screening.
2. Smartphone recordings compared against clinical gold standard statistically.
3. Chronic disease.
4. Human subjects.
5. Peer-reviewed.
6. English language.
7. Year of publication: January 2010–September 2020.
**Exclusion Criteria:**
1. Acute disease.
2. Publications on incomplete or part of research (e.g., editorials, abstracts, workshop/conference summaries, research proposals, descriptive survey, clinical protocols, research methods, literature reviews, conceptual papers).
4. Participants aged < 18 years.
5. Study with less than 15 participants.
6. Systematic survey of apps.
7. Non-human focused (e.g., animals, physical structures, health economic, evaluation of study ethics).
7. Use of non-electronic tools to collect data (e.g., paper-based questionnaire, opinions, viewpoints, health policy).
8. Use of apps for contacting health care providers.
9. Evaluation and development of research tools (e.g., hardware and algorithm improvement studies, guidelines for app developments, assessing the apps, mobile health solutions, and clinical measurement technology to access and analyse secondary data).

**Table 2:** Details of the included studies.

Articles	Study Type	Country	Count	Age	Male (%)	Female (%)
(Katibeh, 2020) [15]	Exp	Iran	2520	61.7 ± 9.5	51.5	48.5
(Aw, 2020) [16]	Obs	Kenya	1650	43–59	37	63
(Treskes, 2019) [17]	Obs	Netherlands	26	62–71	92	8
(Devos, 2019) [18]	Obs	Belgium	15	86.5 ± 5.95	40	60
(Brittz, 2019) [19]	Exp	SA	200	18–55	27	73
(Uchino, 2018) [20]	Obs	Japan	63	24–84	40	60
(Toy, 2016) [21]	Obs	US	50	60.5 ± 10.6	42	58
(BinDhim, 2016) [22]	Obs	AU, Canada, UK, US, NZ	2538	18–75	55	45
(Kim, 2016) [23]	Obs	South Korea	78	44.35 ± 7	-	100

Exp: Experimental; Obs: Observational; SA: South Africa; AU: Australia; UK: United Kingdom; US: United States; NZ: New Zealand.

**Table 3:** Mobile cellular subscriptions (per 100 people) (adapted from [24,25]).

#	Country	Economic Status	Year-Wise Subscription Details		
			2015	2017	2019
1	Australia	Developed	107.7	108.4	110.6
2	Belgium	Developed	113.2	99.5	99.7
3	Canada	Developed	82.6	86.3	92.5
4	Iran	Developing	94.6	107.9	142.4
5	Japan	Developed	125.5	135.5	Not available
6	Kenya	Developing	78.8	85.3	103.8
7	Netherlands	Developed	122.9	120.6	127.3
8	New Zealand	Developed	121.4	136.1	Not available
9	South Africa	Developing	158.9	155.2	165.6
10	South Korea	Developing	116.0	124.6	134.5
11	United Kingdom	Developed	120.3	118.5	Not available
12	United States	Developed	119.1	123.0	Not available

## Data Availability

Source data for all figure(s) and number(s) are provided with the paper and Supplementary Files.

## Supplementary Materials

The following supporting information can be downloaded at: https://www.mdpi.com/article/10.3390/s22103787/s1, Table S1: Medline search strategy.
