# Supplementary material for: Smartphone as a Disease Screening Tool: A Systematic Review
Source: Sensors (Basel). 2022 May 16;22(10):3787. doi: 10.3390/s22103787 (PMC9145643; doi:10.3390/s22103787)
Supplement: Supplementary file 1 [file sensors-22-03787-s001.zip › sensors-1673136-supplementary.pdf]

sensors MDPI

Table S1. Medline search strategy.

#	Searches	Results
1	mhealth OR mobile health OR m-health OR mobile app OR mobile application OR smartphone application OR app OR apps	57,704
2	disease OR illness OR sickness OR condition OR disorder OR health	13,313,920
3	screening OR assessment OR test OR diagnosis	7,500,856
4	1 AND 2 AND 3	14,694
5	Limit 4 to publication years: January 2010 to September 2020	10,217
6	Limit 5 to Academic Journals	10,203
7	Limit 6 to Language: English	10,012
8	Limit 7 to Age Related: All adult (19+ years)	3699
9	Limit 8 to Publication Type: Clinical Study, Clinical trial, Controlled Clinical Trial, Multicentre Study, Observational Study, Randomised Controlled Trial	1213
